# The impact of hypertensive disorders of pregnancy on maternal and perinatal outcomes in Ethiopia: an umbrella review of systematic reviews

**DOI:** 10.3389/fgwh.2025.1571052

**Published:** 2025-07-21

**Authors:** Teketel Ermias Geltore, Simegn Alemu, Tariku Laelago Ersado, Tamiru Beyene Uliso, Abebe Alemu Anshebo, Lakew Lafebo Foto

**Affiliations:** ^1^ Department of Midwifery, College of Medicine and Health Science, Wachemo University Durame Campus, Durame, Ethiopia; ^2^ Department of Nursing, College of Medicine and Health Science, Wachemo University Durame Campus, Durame, Ethiopia; ^3^ Department of Midwifery, College of Medicine and Health Science, Wachemo University, Hosanna, Ethiopia; ^4^ School of Public Health, Institute of Health Science, Bule Hora University, Bule Hora, Ethiopia

**Keywords:** hypertensive disorders of pregnancy, preeclampsia, eclampsia, maternal and perinatal outcomes, Ethiopian pregnant women, systemic review, meta-analysis

## Abstract

**Background:**

Previous systematic reviews and meta-analyses have concentrated on the impacts of hypertensive disorders of pregnancy on maternal and perinatal outcomes in Ethiopia. Still, the evidence has often been inconsistent and inconclusive. Consequently, this study seeks to consolidate the findings regarding the effects of hypertensive disorders during pregnancy on maternal and perinatal outcomes in Ethiopia.

**Methods:**

PubMed, Science Direct, Google Scholar, Africa Journal Online, PsycINFO, Research4Life, and CINAHL from September 15 to 25, 2024. The quality of the methods was assessed using the Assessment of Multiple Systematic Reviews (AMSTAR) tool. The estimates from the included studies were pooled and summarized using random-effects meta-analysis models.

**Results:**

We included five systematic reviews and meta-analyses (SRM) studies with a total of 621,146 pregnant women. The pooled prevalence of hypertensive disorders of pregnancy was 16.56% (95% CI: 13.15–20.02), with a heterogeneity index (I^2^ = 94.17%, *P* = 0.00). Maternal age >35 years, (AOR = 2.29; 95% CI: 2.05, 2.94), previous history of preeclampsia, (AOR = 3.51; 95% CI: 2.26, 5.53), low birth weight (AOR = 2.18; 95% CI: 1.48, 3.01), and alcohol consumption (AOR = 1.84; 95% CI: 1.12, 2.46) were the risk factors and complications of hypertensive disorders of pregnancy.

**Conclusion:**

The higher rate of severe forms of HDP that are associated with significant maternal and perinatal complications is a major concern in Ethiopia. The risk of developing HDP is worse among women who have a history of preeclampsia, maternal age >35 years, alcohol consumption, and its complications, such as low birth weight.

**Systematic Review Registration:**

https://www.crd.york.ac.uk/PROSPERO/view/CRD42024578548.

## Introduction

Hypertensive disorders during pregnancy represent significant public health challenges worldwide (1, 2). Numerous research studies indicate that hypertensive disorders of pregnancy (HDP) are linked to increased rates of morbidity and mortality including intrauterine growth restriction, intrauterine fetal. demise, preterm delivery, birth asphyxia, low birth weight, perinatal mortality, stillbirth, the necessity for admission to neonatal intensive care units, and reduced Apgar scores (3–6). Moreover, numerous risk factors associated with HDP, such as subcapsular hematoma of the liver, abruptio placentae, and disseminated intravascular coagulation, are significantly common among women experiencing hypertension during pregnancy (7).

According to the World Health Organization (WHO), about 15% of pregnancy, childbirth, and postpartum complications are attributed to hypertension (8). HDP significantly contributes to maternal and perinatal morbidity and mortality globally, causing around 30,000 maternal deaths annually, with 10%–15% of these fatalities happening in low- and middle-income countries (9). Of the estimated 2.6 million stillbirths that occur each year, around 16% are affected by hypertension (10).

The prevalence of HDP in Africa is significant, with approximately one in every ten pregnancies being impacted (11). As reported by the WHO, Africa exhibits the highest age-standardized prevalence of hypertension, affecting 46% of adults over the age of 25 (12). The likelihood of developing HDP is heightened in women with a personal or familial history of the condition (11). Studies indicate that a significant proportion of women and newborns in Sub-Saharan Africa face risks associated with preeclampsia and eclampsia, as well as related complications such as placental irregularities and extensive inflammation that arise early in pregnancy, ultimately resulting in endothelial damage (13).

Preeclampsia and eclampsia are responsible for 62,000–77,000 maternal deaths each year (14). Deaths associated with HDP are evident in all categories of these conditions, with eclampsia and pre-eclampsia recognized as the primary factors contributing to mortality (15). The prevalence of preeclampsia in developing countries ranges from 1.8% to 16.7% (16, 17).

A few research on the topic of HDP in Ethiopia has revealed several significant factors that contribute to maternal and neonatal morbidity and mortality (3, 18). In Ethiopia, HDP is the second leading cause of maternal fatalities, premature births, and perinatal mortality as indicated by the 2020 report on maternal perinatal death surveillance and response identified (19).

The occurrence of HDP in Ethiopia varies significantly, with reported rates between 2.3% and 64.1% as indicated by several studies (3, 18, 20, 21) indicating a significant occurrence of HDP in the area. Additionally, the prevalence of pre-eclampsia is reported to be between 1.2% and 19.1% (22). Although previous meta-analyses and systematic reviews (SRMAs) have examined this issue (6, 22–25); the variability in the findings in terms of varying degrees of quality scores, various socio-demographic, determinants, predictors, associated factors, correlates, risk factors, adverse effects of, complications, and consequences of HDP on maternal and perinatal birth outcomes in Ethiopia remains inconclusive that poses challenges for healthcare initiatives and medical management.

Consequently, this study aimed to provide an overview of systematic reviews (SRs) to consolidate existing systematic reviews and meta-analyses regarding the impacts of HDP on maternal and perinatal outcomes, and its determinants among pregnant women in Ethiopia.

## Methods

This umbrella review was conducted using the methodology outlined in the umbrella review of SRMAs studies using the Preferred Reporting Items for Systematic Review and Meta-Analysis (PRISMA) checklist (26) (Supplementary File 1) and the Meta-analysis of Observational Studies guideline (MOOSE) (27) (Supplementary Table S1). The review was undertaken through a systematic synthesis of eligible SRM reports on HDP and adverse outcomes in Ethiopia. To avoid unnecessary duplication of efforts, we performed a thorough examination of the PROSPERO database to locate any recent or current projects pertinent to our subject matter. Our investigation found no ongoing or published articles specifically focusing on this area. Consequently, we have registered this umbrella review with the International Prospective Register of Systematic Reviews (PROSPERO) under the designated registration number CRD42024578548.

### Search strategy

A comprehensive literature review was conducted across prominent electronic databases (including PubMed, Science Direct, Google Scholar, online Africa journal, PsycINFO, Research4life, and CINHALE). Additionally, systematic review databases, including the Cochrane Database of Systematic Reviews were searched from September 15 to 25, 2024 on the impacts of hypertensive disorders of pregnancy on maternal and perinatal outcomes in Ethiopia.

We used Condition, Context, and Population (CoCoPop) questions like; Population: Pregnant women, Setting (context): Ethiopia, Exposure: associated factors or risk factors, HDP, Study design: SRMAs of observational studies. These questions were developed from search keywords and/or Medical Subject Headings (MeSH), which were online Africa journal, PsycINFO, Research4life, and CINHALE). Additionally, systematic review databases, including the Cochrane Database of Systematic Reviews, were searched from September 15 to 25, 2024, combined using the “OR” and “AND” Boolean operators. Two authors (TEG and SA.) independently evaluated the eligibility of all retrieved studies, and any disagreements were resolved through discussion and consensus was reached (Supplementary Table S2).

### Eligibility criteria

#### Inclusion exclusion criteria

A SRMAs were included if it fulfill the following criteria: (i) presented a defined literature search strategy, (ii) appraised its included studies using a relevant tool, (iii) SRMAs that utilized observational study designs (cross-sectional, cohort, and case-control) that assessed the prevalence of HDP (iv) followed a standard approach in pooling studies and providing summaries estimates, (v) included research synthesis of all quantitative design (vi) published works reporting on the measures of interest, were in English, and with no restrictions on the year of publication. Exclusions applied to narrative reviews, editorials, correspondence, abstracts, methodological studies, and literature reviews that did not present a clear research topic, search strategy, or defined article selection method.

### Study screening and selection

Initially, two researchers assessed the studies based on defined inclusion and exclusion criteria. They commenced their evaluation by reviewing the titles and abstracts of the studies identified in the databases. Subsequently, the chosen studies were subjected to a comprehensive full-text screening. The PRISMA flow diagram was employed to record the justifications for the inclusion or exclusion of each study. A compilation of studies deemed suitable for data extraction in the umbrella review was created (Table 1).

**Table 1:** Characteristic of the included reviews studies on impacts of hypertensive disorders of pregnancy and its determinants in Ethiopia, 2024.

Authors (year)	Review objective	Search strategy	Included studies	Reported prevalence	Sample size	Risk of Bias	Author conclusion	AMSTAR quality
Berhe et al. (2018)	Prevalence of hypertensive disorders of pregnancy in Ethiopia	MEDLINE, PubMed, EMBASE, HINARI, Google Scholar and the African Journals Online (AJOL).	17 (4 were CC and 13 were CS	6.07 (4.83–7.31), I^2^ = 99.4%	258,602	The quality of included studies were appraised clearly	The prevalence of hypertensive disorders of pregnancy is high in Ethiopia. The problem is more common among older pregnant women (>35 years old).	9
Kassa et al. (2023)	Prevalence of pre-eclampsia and its determinants in Ethiopia	Google Scholar, Pub-med/ Med-line, Scopus, Web of Sciences, and grey literature.	30 (15 CS,13 CC and 2 cohort	11.51 (8.41–14.61), I^2^ = 99.8%	31,201	The quality of included studies were appraised clearly	Prevalence of pre-eclampsia was high. Pre-eclampsia is associated with maternal age >35 years, being a housewife, having a history of preeclampsia, having a history of chronic hypertension, having a family history of hypertension, having diabetes mellitus, drinking alcohol during pregnancy, and having multiple pregnancies	11
Mersha et al. (2019)	Maternal and perinatal outcomes of pregnancies complicated by hypertension in Ethiopia	MEDLINE, Scopus, PubMed, Science Direct, and Google Scholar.	13 (11 CS and 2 cohort)	19.7 (14.25, 25.5), I^2^ = 90.05%	5,894	The quality of included studies were appraised clearly	One in four of pregnancies complicated by hypertensive disorder end up in perinatal death in Ethiopia. HELLP syndrome, placental abruption, pulmonary edema, renal damage, prematurity, perinatal asphyxia, and low birth weight were also commonly reported	10
Tesfa et al. (2020)	Prevalence and risk factors of hypertensive disorder of pregnancy in Ethiopia	PubMed, Scopus, Google Scholar, Hinari, and African Journals Online	34 (13 CC, 20 CS and 1 cohort)	5.78 (4.95–6.62), I^2^ = 92.5%	320,942	The quality of included studies were appraised clearly	Prevalence of hypertensive disorder of pregnancy is relatively higher compared with the previous reports. Maternal age ≥35 years, twin pregnancy, previous history of preeclampsia, family history of hypertension, family history of diabetes mellitus, body mass index ≥25, alcohol consumption, urinary tract infection, lack of fruits and vegetables during pregnancy were risk factors of hypertensive disorder of pregnancy	10
Getaneh et al. (2020)	Impact of pregnancy induced hypertension on low birth weight and its association in Ethiopia	PubMed/Medline, EMBASE, CINAHL, Cochrane library, Google, Google Scholar and local shelves.	25 (15 CS, 5 cohort and 5 CC	39.7 (33.3–46.2), I^2^ = 89.4%	4,507	The quality of included studies were appraised clearly	Prevalence of low birth weight among women who had pregnancy induced hypertension was more than two times higher than the pooled estimate of low birth weight among all reproductive aged women.	10

CC, case control; CS, cross sectional.

### Outcomes measures

This umbrella review highlights two primary outcomes. The first outcome is the prevalence of hypertensive disorders of pregnancy (HDP) among expectant mothers.

Hypertension during pregnancy is recognized by the American College of Obstetricians and Gynecologists (ACOG) when the systolic blood pressure (SBP) reaches 140 mm Hg, the diastolic blood pressure (DBP) is 90 mm Hg, or both measurements are elevated, ideally confirmed on two separate occasions or at least four hours apart (28). Furthermore, the International Society for the Study of Hypertension in Pregnancy (ISSHP) has updated the classification of hypertensive disorders of pregnancy (HDP) to encompass chronic hypertension, white coat hypertension, masked hypertension, gestational hypertension, and pre-eclampsia (29).

Eclampsia is characterized by the occurrence of convulsions; a DBP of 90 mm Hg or higher after 20 weeks of pregnancy; proteinuria of 2+ or greater; and signs and symptoms indicative of severe pre-eclampsia (30).

Pre-eclampsia pertains to women who experience both hypertension and proteinuria during pregnancy (31).

Gestational hypertension is defined as an increase in DBP to 90 mm Hg or more without proteinuria in a previously normotensive non-proteinuric pregnant woman (31).

Chronic hypertension in pregnancy is diagnosed based on the presence of hypertension at the first “booking visit” before the 20th week of pregnancy in the absence of trophoblastic disease or at any stage of pregnancy in women with established chronic hypertension, or which persists for more than 42 days following delivery (31).

The second outcome seeks to investigate the predictors, determinants, associated factors, correlates, and influencing factors of HDP. By synthesizing findings from various studies, the review aims to provide a thorough understanding of the prevalence and determinants of HDP within this demographic.

### Data extraction

Data from the included studies were extracted using a standardized data extraction form, developed in an excel sheet. For each study, the following data were extracted: identification data.

(a) (First author's last name and publication year), (b). Measure of magnitude (prevalence for HDP) (c) Factors associated with HDP (odds ratio or relative risk) with 95% confidence intervals, (d) Number of studies included, (e) Complications of HDP, (f) Total number of samples included, (g) Publication bias assessment methods and scores, quality assessment methods and scores, (h) Data synthesis methods (random or fixed-effects model), (i) Review aim, (j) The authors’ main conclusion of the SRM study.

The Endnote citation manager (version X8, for Windows; Thomson Reuters, Philadelphia, PA, USA) was applied to import the retrieved studies.

### Quality assessment

Each study included in the analysis was subjected to a comprehensive evaluation utilizing the Assessment of Multiple Systematic Reviews (AMSTAR) tool (32), which comprises 11 questions aimed at assessing both methodological and evidential integrity. Quality ratings were assigned on a scale from 0 to 11, with scores categorized as high (8–10), medium (4–7), or low (<3). In accordance with Cochrane guidelines, we conducted a further assessment of the overall strength and quality of the studies using the GRADE tool, which evaluates five risk factors: bias, consistency, directness, accuracy, and publication bias. The quality rating indicates a deterioration in the quality assessment (33) (Table 2). The evaluation process was carried out by three authors (TEG, LLF, and SA) who examined various elements of each study, including methodological quality, sample selection, sample size, comparability, outcomes, and statistical analysis. In cases where disagreements arose among the three authors, two additional authors (TLE and TBU) were consulted to promote discussion and reach a resolution.

**Table 2:** Summary of results and quality of evidence assessment using the GRADE approach.

Outcome measures	Summary of findings		Quality of evidence assessment (GRADE)					
	No. of participants/number of meta analyses (included studies)	Effect size (95% CI)	Risk of bias^a^	Inconsistency ^b^	Indirectness ^c^	Imprecision ^d^	Publication bias^e^	Quality of evidence^f^
The prevalence of HDP among expectant mothers	621,146/5	16.59 (13.2, 20.0)	Not serious	Serious	Not serious	Not serious	Not serious	High

^a^ Risk of bias based on the AMSTAR results.

^b^ Downgraded if there was a substantial unexplained heterogeneity (I^2^ > 50%, *P* < 0.10) that was unexplained by meta-regression or subgroup analyses.

^c^ Downgraded if there were factors present relating to the participants, interventions, or outcomes that limited the generalizability of the results.

^d^ Downgraded if optimal information size was not met, or the 95%CI include the null value lower and upper bounds of the 95% CI were <0.95 and >1.05, respectively.

^e^ Downgraded if there was an evidence of publication bias using funnel plot.

^f^ Since all included studies were meta-analyses, the certainty of the evidence was graded as high for all outcomes by default and then downgraded based on pre specified criteria. Quality was graded as high, moderate, low, very low.

### Data synthesis

The included SRMA estimations were compiled using qualitative and quantitative methods. The range of estimations for HDP's magnitude, associated factors, and complications was shown, and a summary (pooled) estimate was computed in cases where two or more estimates were given. Higgins' I^2^ statistics were used to determine the degree of heterogeneity between studies, which guided the choice of meta-analysis methodology (34). According to Higgins et al, I^2^ < 49%, 50–75, and >75%, respectively, indicate low, moderate, and high degrees of heterogeneity. Because of the substantial variations within and between studies, the random-effects model was used to produce the pooled prevalence estimates (34). Because just five studies were included, it was not possible to assess publication bias. Usually, at least ten researches are needed to assess publication bias (35). STATA version 17.0 was used to conduct quantitative analysis. An overview of the HDP predictors and their corresponding odds ratios was created.

### Ethical consideration

Because the study used data from SRMAs, it was not essential to get participants' agreement or ethical approval.

## Results

### Study selection

**Figure 1:**
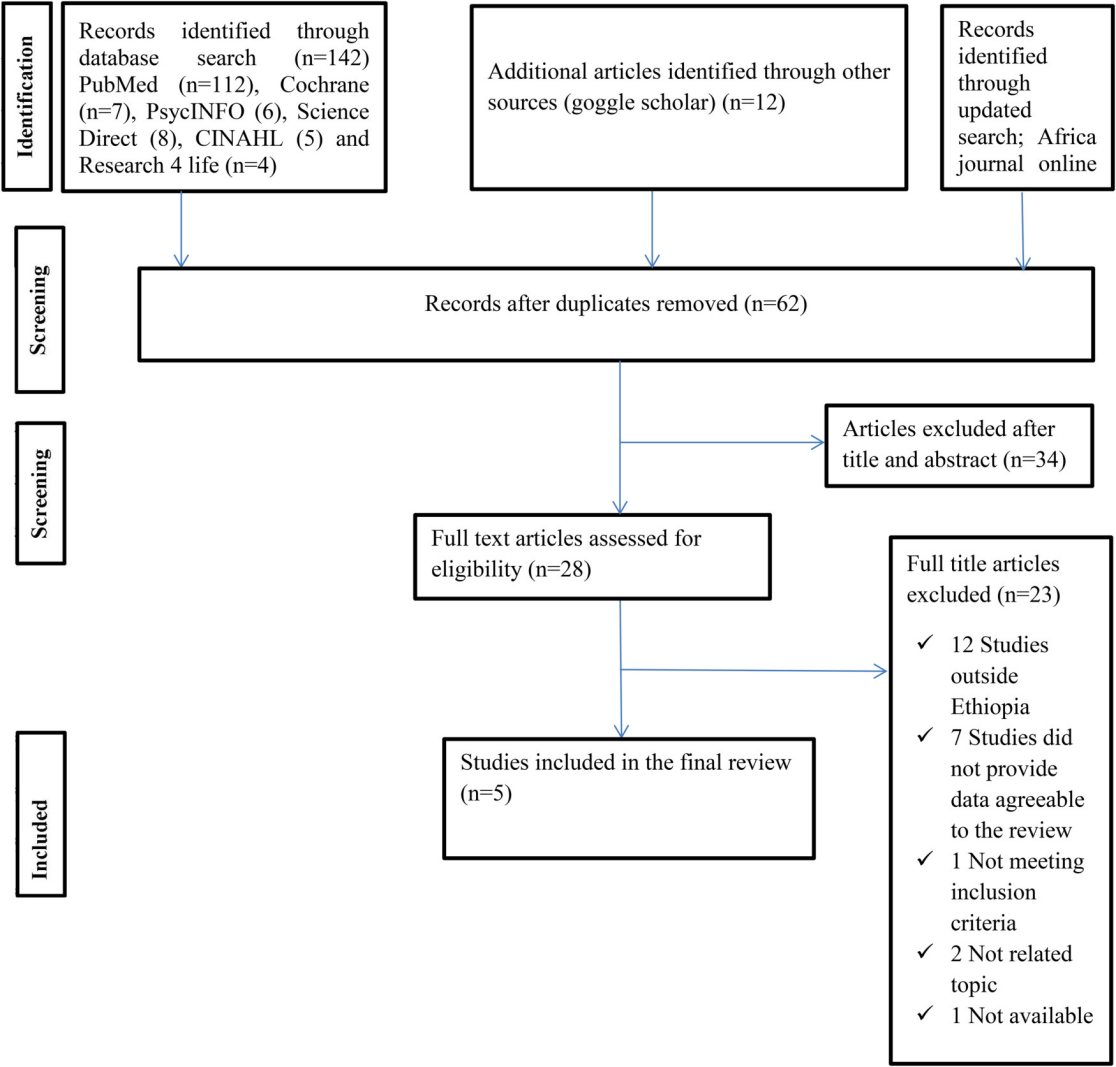
PRISMA flow chart displays the article selection process for umbrella review on impacts of hypertensive disorders of pregnancy and its determinants in Ethiopia.

Initially, our database search resulted in 156 articles. Subsequently, after eliminating 94 duplicate entries, we were left with 62 records. A review of the titles and abstracts led to the exclusion of 34 articles. The remaining 28 articles were then assessed for eligibility. Ultimately, 23 articles were excluded for several reasons, such as being conducted outside of Ethiopia, lacking relevant data for the review, failing to meet inclusion criteria, addressing unrelated topics, or being unavailable. In conclusion, five studies were included in the review (6, 22–25) as shown in (Figure 1).

### Characteristics of included studies

This umbrella review included five systematic reviews and meta-analyses (6, 22–25). Seventy-four cross-sectional, thirty-two case-control and 10 cohort studies were included in the review, comprising the sample size of 621,146 pregnant women. The sample size per each systematic review and meta-analysis ranges from 4,507 to 320,942 and the number of primary studies included per each systematic review and meta-analysis ranges from 13 (6) to 30 (24). From five of the SRMA studies included in this umbrella review, two SRMA were published in 2020 (24, 25) and one each was published in 2018 (22), 2019 (6), and 2023 (23). All included SRMA studies assessed both the prevalence and determinants of HDP in Ethiopia. Among those including reviews, the prevalence of all forms of HDP ranges from 6.07 (4.83%, 7.31%, I^2^ = 99.4%) (22) to 6.82 (5.90, 7.74, I^2^ = 99.2%) (24), and the prevalence of pre-eclampsia ranges from 4.74% (3.99, 5.49, I^2^ = 95.9%) (24) to 11.51% (8.41, 14.61, I^2^ = 99.8%) (23) (Table 1).

### Description of primary included studies

This umbrella review highlighted the redundancy of primary studies present in each systematic review and meta-analysis. A total of 116 studies were included in the systematic review and meta-analysis, as shown in (Table 1). Among these, 18 studies were found to overlap during the critical appraisal of the overall systematic review and meta-analysis, while the others did not exhibit any overlap. Notably, a maximum of six studies were featured in two or more systematic reviews and meta-analyses (36–41). These studies were reviewed by Kassa et al. (23) and Tesfa et al. (24). Besides this, in the reviews conducted by Berhe et al. (22) and Tesfa et al. (24) three pocket studies were incorporated (29, 42, 43). Similarly, two studies each (44, 45) and (4, 46) were included in the review done by Berhe et al. (22), Kassa et al. (23) and Tesfa et al. (24); and Berhe et al. (22), Kassa et al. (23), Mersha et al. (6), Tesfa et al. (24), and Getaneh et al. (25) respectively. In addition, one study each (47–52), was included in the review conducted by Berhe et al. (23), Mersha et al. (6), Tesfa et al. (24), and Getaneh et al. (25); Mersha et al. (6) and Getaneh et al. (25); Mersha et al. (6), and Berhe et al. (22), Kassa et al. (23), and Tesfa et al. (24) (Table 3).

**Table 3:** Primary studies included in the systematic review and meta-analysis on impacts of HDP and its determinants among pregnant women Ethiopia 2024.

Review studies	Berhe et al. (23)	Kassa et al. (24)	Mersha et al. (25)	Tesfa et al. (26)	Getaneh et al. (27)
Primary studies					
Gudeta et al. (30)		^a^		^a^	
Belay and Wuded et al. (31)		^a^		^a^	
Hinkose et al. (32)		^a^		^a^	
Legesse et al. (33)		^a^		^a^	
Wodajo et al. (34)		^a^		^a^	
Mikie et al. (35)		^a^		^a^	
Hailu and Kebede et al. (36)	^a^				^a^
Gaym et al. (37)	^a^				^a^
Akililu et al. (38)	^a^				^a^
Tessema et al. (39)	^a^	^a^		^a^	
Shagze et al. (40)	^a^	^a^		^a^	
Waganew et al. (41)	^a^	^a^	^a^	^a^	^a^
Vata et al. (42)	^a^	^a^	^a^	^a^	^a^
Seyome et al. (29)	^a^		^a^	^a^	^a^
Obsa et al. (43)			^a^		^a^
Wolde et al. (44)			^a^		^a^
Tefera et al. (45)	^a^	^a^		^a^	^a^
Selamawit and Sisay et al. (46)			^a^	^a^	

NB, the mark.

^a^ Indicates the number of primary studies included in the systematic review and meta- analysis.

### The methodological quality of the included SRM studies

An evaluation of the methodological quality of systematic reviews and meta-analyses (SRMA) using the AMSTAR tool showed quality scores ranging from 9 to 11 points, with an average score of 9.4 points, signifying a high standard of quality.

Each SRMA performed a comprehensive search, utilized appropriate methods for synthesizing results, and clearly disclosed potential sources of support in both the systematic review and the individual studies. Furthermore, employing the GRADE instrument, all qualitative effects were assigned a high rating (Table 4).

**Table 4:** Methodological quality of the included SRM studies based on AMSTAR tool in Ethiopia, 2024.

AMSTAR tool	Authors				
	Berhe al.	Kassa et al.	Mersha et al.	Tesfa et al.	Getaneh et al.
Priori design provided	No	Yes	Yes	Yes	Yes
Duplicate study selection and data extraction	No	Yes	No	Yes	Yes
Search comprehensiveness	Yes	Yes	Yes	Yes	Yes
Inclusion of grey literature	Yes	Yes	Yes	Yes	Yes
Included and excluded studies provided	Yes	Yes	Yes	Yes	Yes
Characteristics of the included studies provided	Yes	Yes	Yes	Yes	Yes
Scientific quality of the primary studies assessed and documented	Yes	Yes	Yes	No	No
Scientific quality of included studies used appropriately in formulating conclusions	Yes	Yes	Yes	Yes	Yes
Appropriateness of methods used to combine studies’ findings	Yes	Yes	Yes	Yes	Yes
Conflict of interest	Yes	Yes	Yes	Yes	Yes
Likelihood of publication bias was assessed	Yes	Yes	Yes	Yes	Yes
Total (11)					

### Prevalence of HDP among pregnant women in Ethiopia

**Figure 2:**
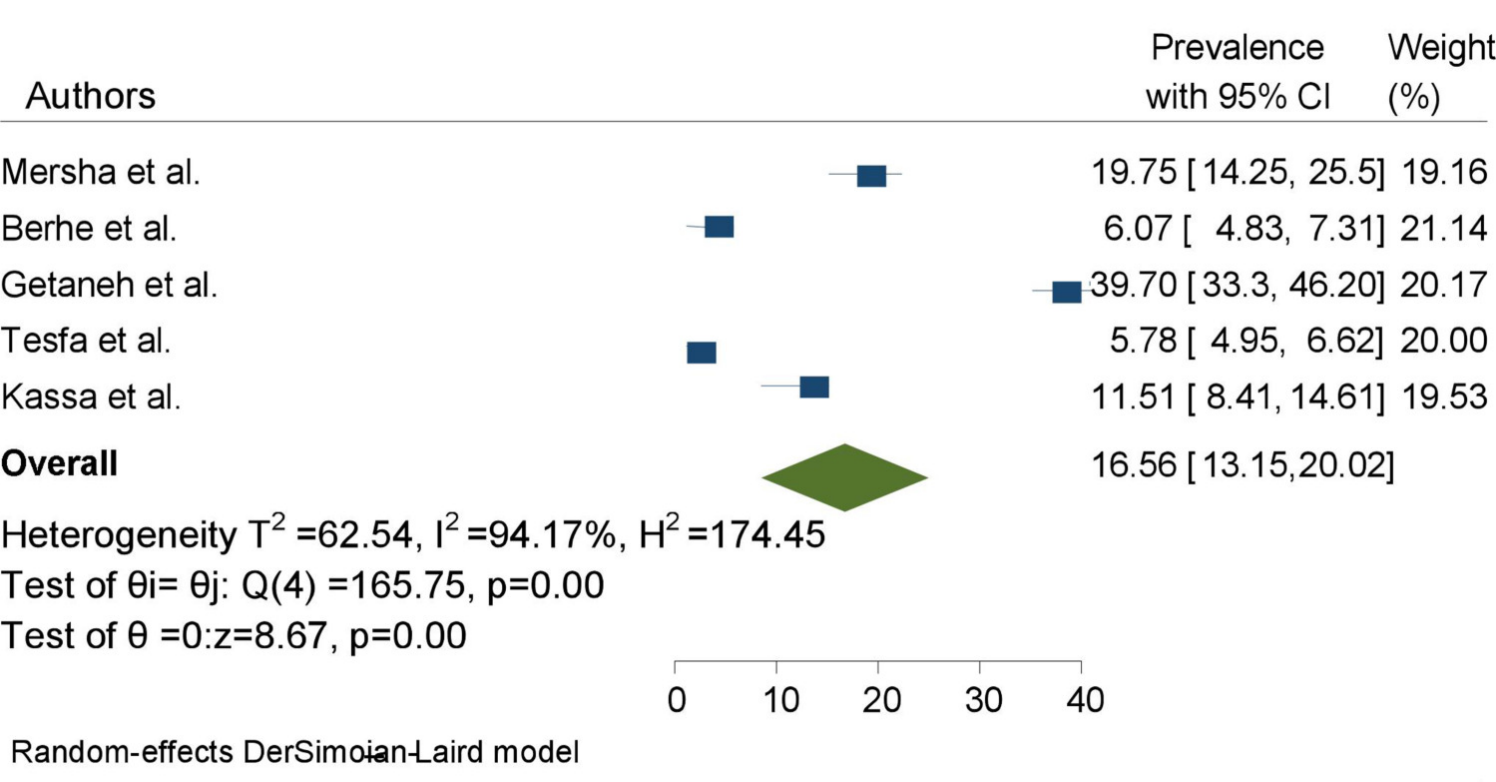
The pooled prevalence of impacts of hypertensive disorders of pregnancy among pregnant women in Ethiopia.

The pooled prevalence of HDP among pregnant women in Ethiopia was 16.56%, (95% CI: 13.15–20.02), with the heterogeneity index (I^2^ = 94.17%, *P* = 0.00) showing considerable heterogeneity of different reviews (I^2^ > 75%). Hence, we have used the random effect model to resolve the concern of heterogeneity among the included reviews (Figure 2).

### Subgroup analysis

The subgroup analysis focusing on sample size revealed that the sample size less than 10,000 had the highest prevalence of use of HDP 27.7% (95% CI, 18.81, 30.49), whereas sample size greater than10,000 had the lowest prevalence 5.43% (95% CI, 7.50, 9.62) (Supplementary Table S3).

### Sensitivity analysis

We performed a comprehensive examination of the sources of heterogeneity through a leave-one-out sensitivity analysis. This analysis revealed that the exclusion of any single study from the overall evaluation did not significantly alter the estimated average prevalence. The average prevalence consistently remained within the 95% confidence interval of the overall average prevalence calculated when all studies were included. Consequently, no individual study had a significant effect on the average prevalence. Additionally, the sensitivity analysis demonstrated that the removal of each study individually resulted in an average prevalence of 16.56%, with a 95% confidence interval ranging from 13.15, 20.02, as shown in (Supplementary Table S4).

### Risk factors and complication of HDP

The included SRMA studies (6, 22–25) in this umbrella review investigated various factors associated with HDP and its complications. These studies reported factors such as maternal age greater than 35 years, alcohol consumption during pregnancy, prior history of preeclampsia, and low birth weight.

**Figure 3:**
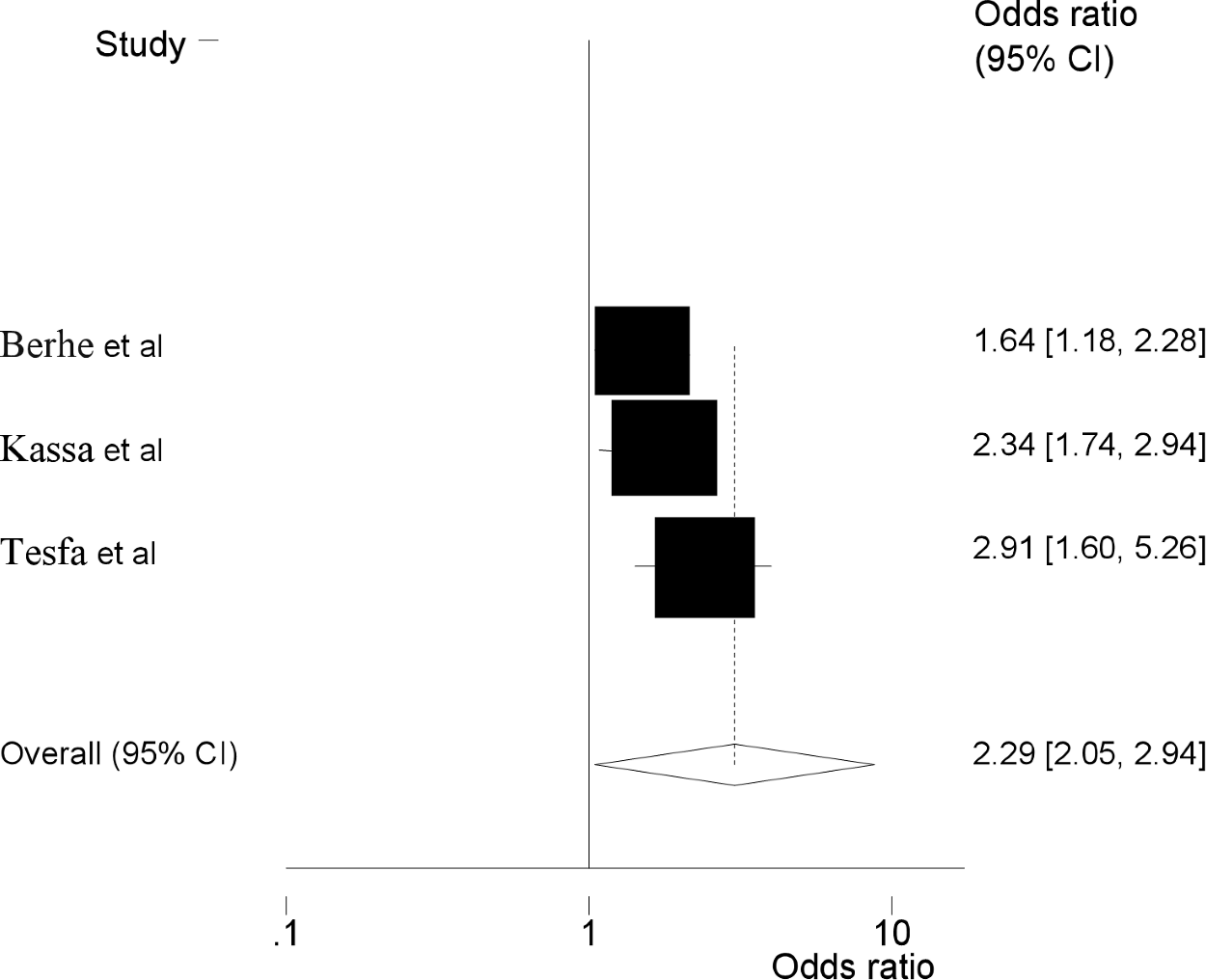
Umbrella review about the pooled effects of maternal age as risk factor for hypertensive disorders of pregnancy.

**Figure 4:**
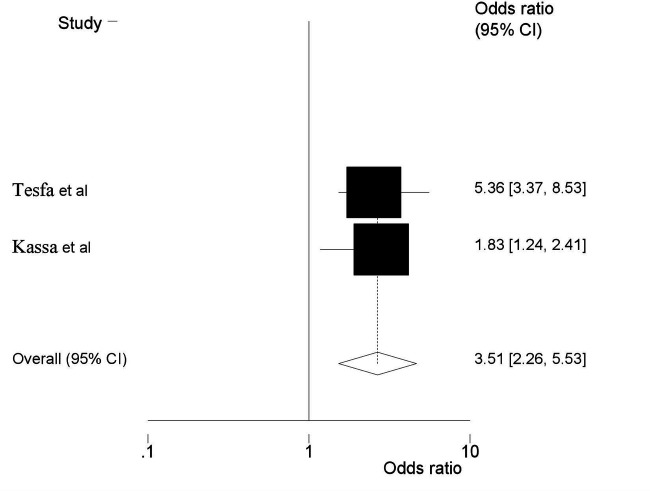
Umbrella review about the pooled effects of previous history of preeclampsia as risk factor for hypertensive disorders of pregnancy.

Three SRMA (22–25) reported that maternal age had a significant association with the prevalence of HDP. Women whose age is >35 years were 2.29 times more likely to develop HDP than women aged 20–34 during their pregnancy (AOR = 2.29; 95% CI: 2.05, 2.94) (Figure 3). This umbrella review included two SRMA (23, 24) revealed that previous history of preeclampsia is a risk factor for HDP. In this meta-analysis, women with a history of pre-eclampsia were shown to develop pre-eclampsia, with the likelihood of its occurrence almost four times higher in women with no history of pre-eclampsia, and the pooled odd ratio showed that the association was statistically significant (AOR = 3.51, 95% CI = 2.26, 5.53) (Figure 4).

**Figure 5:**
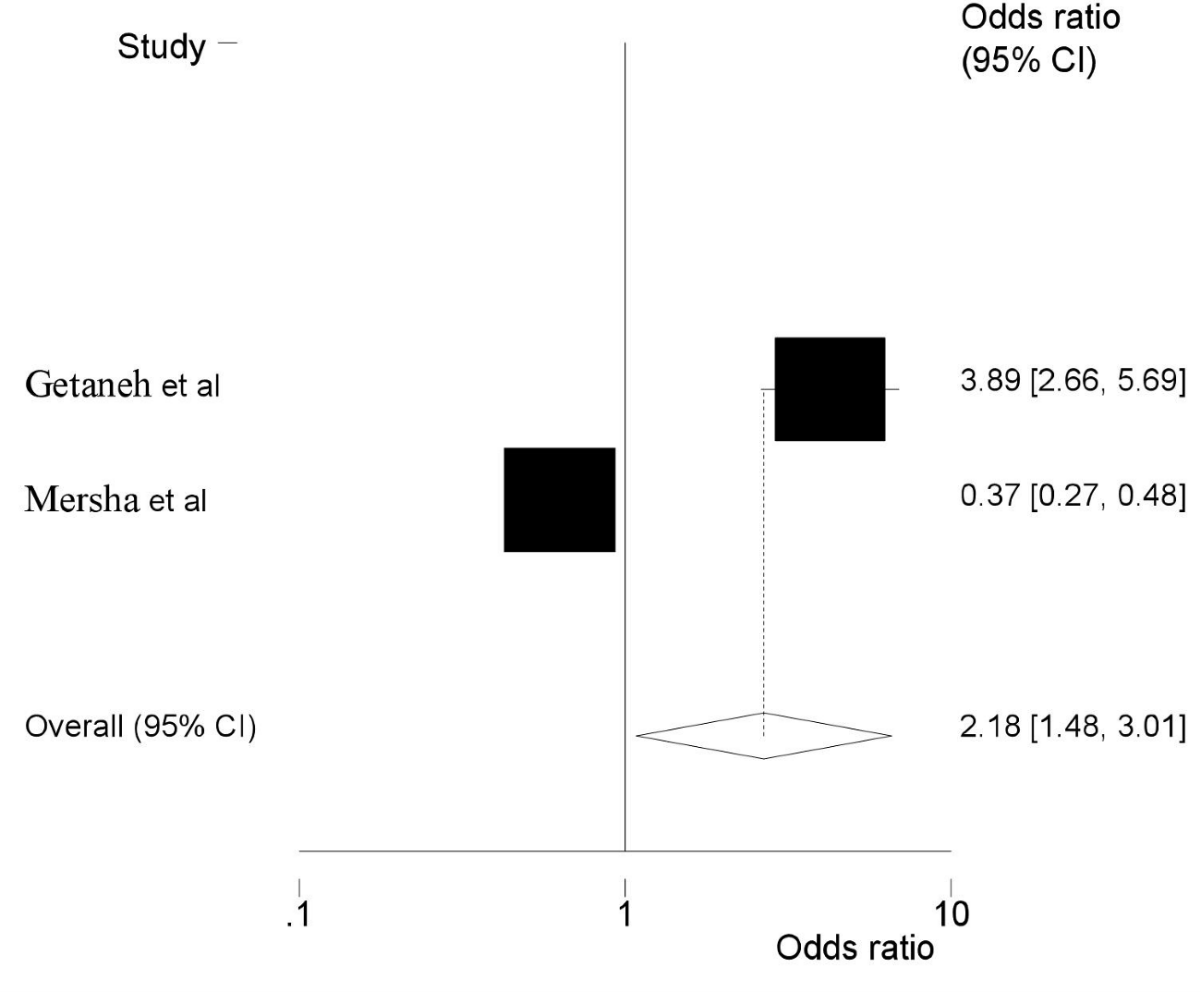
Umbrella review about the pooled effects of low birth weight from hypertensive disorders of pregnancy. Forest plot showing odds ratios with 95% confidence intervals for two studies.

**Figure 6:**
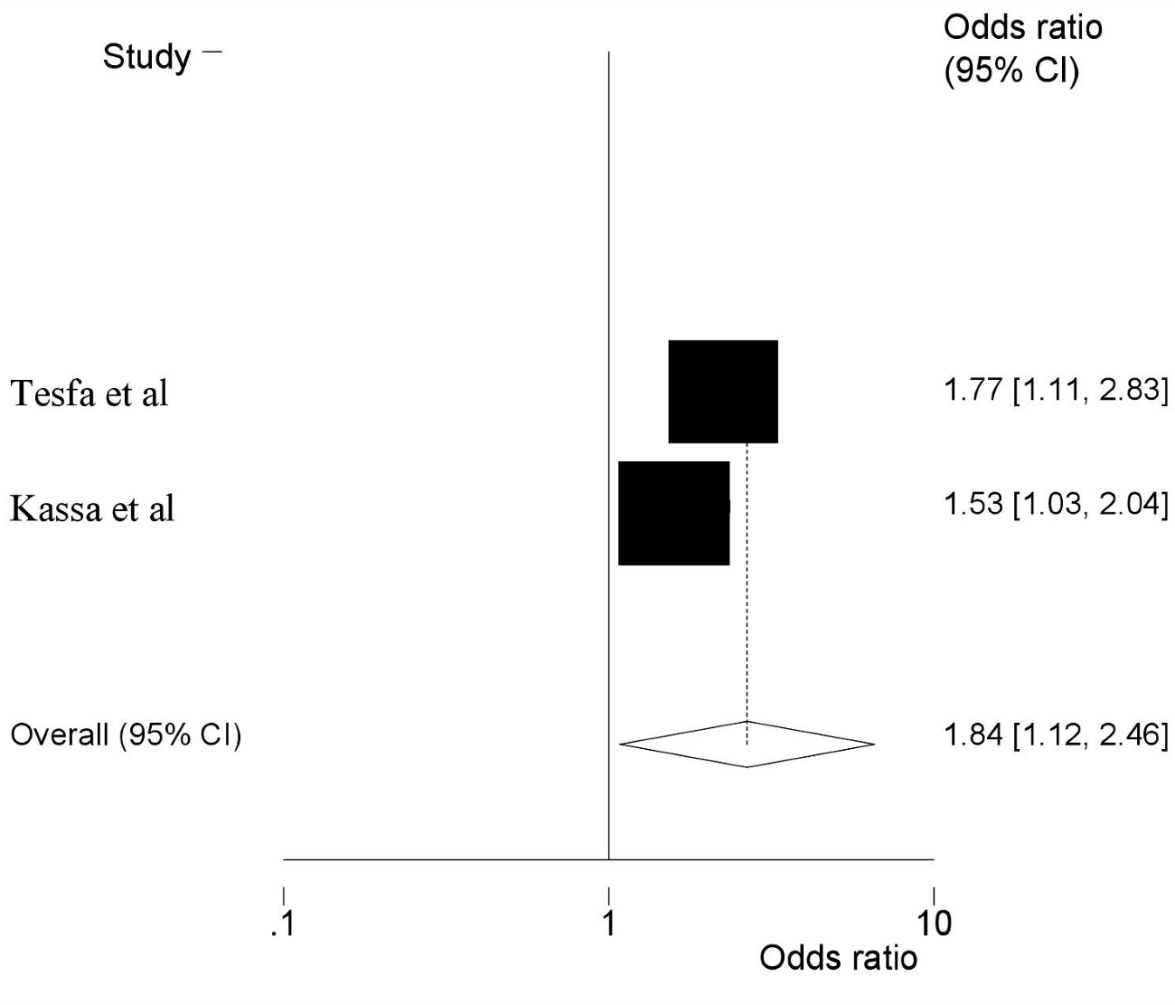
Umbrella review about the pooled effects of alcohol consumption as risk factor for hypertensive disorders of pregnancy.

Moreover, two SRMA (6, 25) showed that low birth weight had a significant association with HDP. Accordingly, the odds of HDP were almost twice as high as among women who had HDP compared to their counterparts. (AOR = 2.18, 95% CI: 1.48, 3.01) (Figure 5). Furthermore, two additional SRMA (23, 24) reported that there was an association between alcohol consumption and HDP. In the current umbrella review, alcohol consumption during pregnancy had revealed the odds of developing HDP to be 1.84 times more likely compared with the women who did not drink alcohol (AOR = 1.84, 95% CI: 1.12, 2.46) (Figure 6).

## Discussion

Five SRM reports about HDP in Ethiopia have been published so far. Most agree that these SRM researches offer solid proof to support health programs in decision-making. However, it could become difficult for people looking for information when individual reviews increase (53). Thus, this umbrella review was conducted to synthesize the findings from the five SRM studies on HDP into a comprehensive document. Furthermore, factors such as maternal age >35 years, previous history of preeclampsia, low birth weight, and alcohol consumption were identified as statistically significant in determining the impacts of HDP in Ethiopia.

The comprehensive review of the five selected systematic review and meta-analysis studies regarding the HDP in Ethiopia revealed a summary estimate of 16.56% (95% CI: 13.15, 20.02). This finding was higher compared to the finding from the global prevalence (5.2%–8.2%) (54), the study conducted in China, which reported a prevalence of (5.2%) (55), in the United States (6%–8%) (56, 57), in Australia (8.2%) (58), and meta-analysis done in Africa which was 10% (11).

The higher prevalence in our country may be attributed to the inability to incorporate American College of Obstetricians and Gynecologists (ACOG) guidelines where; ACOG and other international agencies updated diagnostic guidelines a decade ago to align HDP signs and symptoms with those most closely associated with maternal death in low and middle-income countries (59). The overestimation of the prevalence of HDP in Ethiopia may stem from several factors: many of the studies included in the review were hospital-based, which might not adequately represent the general population, health system access, variations in diagnostic criteria and data collection methods among studies and socioeconomic status and health system limitations (60). Delays in attending antenatal care can lead to missed opportunities for addressing potential complications, which can negatively affect both maternal and fetal health (61); even when the number of contacts is many, the quality of ANC care is often inadequate, as blood pressure and proteinuria measurement may not be regularly accessible (62).

In addition, populations in developed nations tend to be better educated, maternity services are likely to be used more frequently than in Ethiopia, and health systems are typically more developed and equipped with qualified healthcare professionals. Integrating culturally appropriate policies to identify and treat women with HDP early in programs aimed at lowering mother and child mortality should be the top priorities in our nation, as a considerable percentage of severe forms of HDP contribute to the high rates of adverse maternal and perinatal outcomes in rural areas. Raising public awareness of the necessity of pregnant women receiving at least one consultation during the first half of their pregnancy for a routine blood pressure check and the encouragement of appropriate systematic blood pressure measurement and promotion of adequate calcium intake through locally available, calcium-rich foods are also advisable. Besides, ensuring timely referrals to nearby health facilities, and strengthening maternity waiting homes should also be organized and supported. Moreover, encouraging a balanced diet and consistent physical exercise is vital for minimizing risks. In addition, enhancing healthcare facilities for prompt interventions during high-risk pregnancies is imperative. This umbrella review showed that maternal age had a significant association with the prevalence of HDP. Women whose age is >35 years were 2.29 times more likely to develop HDP than women aged 20–34 during their pregnancy. This finding is supported by a study conducted on pregnant women in China (55) and Kenya (63). A persistently increased lipid profile, high-density lipid cholesterol, and a higher risk of vascular damage in this age group relative to young females may be the cause of the increased risk in older mothers (64). There was also a 4% increase in the rate of late pre-eclampsia and gestational hypertension for every year over the age of 32 (65). Early screening and identification for those women whose ages are greater than 35 years are crucial tasks to HDP. Moreover, age is one of the non-modifiable risk factors for HDP. As a result, this is the fact that the cardiovascular system is strongly affected by aging; besides, aging causes structural and functional changes in the blood vessels that may lead to maternal morbidity and mortality (66). Maternal age exceeding 35 years is a notable risk factor for hypertensive disorders during pregnancy in Ethiopia, shaped by both biological and socio-economic influences. Mothers in this age bracket are at an increased risk for conditions such as preeclampsia and gestational hypertension, which are often exacerbated by existing health problems. Additionally, this demographic experiences elevated rates of chronic illnesses, including diabetes and hypertension, which can complicate pregnancy. Furthermore, restricted access to healthcare in Ethiopia may lead to adverse maternal and fetal outcomes.

The current review revealed a significant association between HDP and previous history of preeclampsia. In this meta-analysis, women with a history of pre-eclampsia were shown to develop pre-eclampsia, with the likelihood of its occurrence almost four times higher in women with no history of pre-eclampsia. This finding is supported by a study conducted in China (67), and Sub-Saharan Africa (68). This could have happened because of genetic factors that contribute to the physiologic predisposition to HDP.

The umbrella review also found that HDP has a significant impact on fetal well-being. Specifically, low birth weights were almost twice as high among women who had HDP compared to their counterparts. This finding is supported by a study conducted in Ghana (69), China (1) and Haiti (70). Hypertensive disorders in pregnancy can restrict blood flow to the placenta, limiting nutrients and oxygen for the fetus, which may result in low birth weight (71). To mitigate these risks, it is essential to manage blood pressure effectively. Consistent prenatal care, a nutritious diet, suitable physical exercise, and medication when necessary can assist in sustaining healthy blood pressure levels and promoting optimal fetal growth. The current review revealed a significant association between HDP and alcohol consumption during pregnancy in which the odds of developing HDP were 1.84 times more likely in alcohol users compared with the women who did not drink alcohol. This may be the result of uteroplacental malperfusion, which can happen during pregnancy and may also play a role in the association between alcohol intake and HDP (72). Furthermore, drinking alcohol has an impact on the central nervous system, which raises heart rate and influences peripheral vascular effects (73). Socioeconomic difficulties and insufficient job opportunities may drive pregnant women to resort to alcohol as a coping mechanism. To effectively address alcohol consumption among pregnant women, it is crucial to confront these underlying issues by fostering gender equality, enhancing economic prospects, and establishing community support networks that challenge harmful cultural practices (74). Additionally, societal norms and expectations frequently influence the behavior of pregnant women, leading to restricted access to resources and opportunities that could aid in decreasing alcohol use.

### Implications of the study

This research provides current and succinct evidence regarding the impacts of HDP on maternal and perinatal outcomes in Ethiopia. It serves as a valuable resource for program developers and implementers across different sectors, such as government and non-governmental aim to ensure the safety of a pregnant woman and her baby. Furthermore, the study highlights the importance of incorporating the American College of Obstetricians and Gynecologists (ACOG) guidelines which are designed for low and middle-income countries. The findings underscore the importance of preventing HDP maternal and prenatal birth outcomes through addressing public awareness of the necessity of pregnant women receiving at least one consultation during the first half of their pregnancy for a routine blood pressure check and promotion of adequate calcium intake through locally available, calcium-rich foods.

### Strengths and limitations of the study

The current review possesses several strengths, notably the rigorous efforts made to mitigate bias through comprehensive searches across various databases. To our knowledge, there has not been a thorough evaluation in the form of an umbrella review regarding the topic of HDP in Ethiopia, despite the existence of numerous empirical studies and specific SRMA investigations. Additionally, we utilized the AMSTAR-2 tool to evaluate the methodological quality of the reviews and, we examined the primary articles within the SRMA reports to detect any overlapping data among the included SRMA studies. Nonetheless, certain limitations are present. Firstly, the small number of studies included presents a challenge. Additionally, despite considerable efforts to address the issue, heterogeneity was still apparent among the included studies, indicating that there were unresolved discrepancies in methodologies or populations. Secondly, the nature of meta-analysis, which relies on aggregated data, limits the identification of confounding factors. This may have affected the pooled estimate. Therefore, any interpretations must consider this limitation.

## Conclusions

The incidence of all forms of HDP is notably high. Consequently, it is essential to enhance awareness initiatives aimed at promoting early antenatal care (ANC) attendance through existing platforms. This includes mobilizing communities to understand the significance of early ANC visits and follow-ups, raising awareness about critical warning signs during pregnancy, and ensuring timely referrals to nearby health facilities. Furthermore, it is essential to provide training for healthcare professionals and to incorporate HDP screening into standard ANC. Future research should focus on prospective studies that comprehensively document blood pressure levels, proteinuria, diagnostic symptoms, laboratory tests for organ dysfunction, and perinatal outcomes to accurately determine the incidence of HDP. Policymakers should implement suitable policies to recognize and address the needs of women with HDP, thereby fostering a more supportive atmosphere for expectant mothers, which will ultimately enhance the outcomes for both mothers and their offspring.

## Data Availability

The original contributions presented in the study are included in the article/Supplementary Material, further inquiries can be directed to the corresponding author.

## References

[1] Bridwell M Handzel E Hynes M Jean-Louis R Fitter D Hogue C Hypertensive disorders in pregnancy and maternal and neonatal outcomes in Haiti: the importance of surveillance and data collection. BMC Pregnancy Childbirth. (2019) 19(1):208. 10.1186/s12884-019-2361-0 31221123 PMC6585002

[2] UNICEF, WHO, World Bank Group, United Nations. Levels and Trends in Child Mortality Report 2018.

[3] Asseffa NA Demissie BW. Perinatal outcomes of hypertensive disorders in pregnancy at a referral hospital, southern Ethiopia. PLoS One. (2019) 14(2):e0213240. 10.1371/journal.pone.0213240 30817780 PMC6394918

[4] Wagnew M Dessalegn M Worku A Nyagero J. Trends of preeclampsia/eclampsia and maternal and neonatal outcomes among women delivering in Addis Ababa selected government hospitals, Ethiopia: a retrospective cross-sectional study. Pan Afr Med J. (2016) 25(Suppl 2):12. 10.11604/pamj.supp.2016.25.2.9716 28439336 PMC5390070

[5] Mi A Mm K Fa S Sa A Ar M Hossain A. Evaluation of maternal and perinatal outcome in pregnancy induced hypertension. Med Col J. (2019) 4(1). 10.1111/obr.12293

[6] Mersha AG Abegaz TM Seid MA. Maternal and perinatal outcomes of hypertensive disorders of pregnancy in Ethiopia: systematic review and meta-analysis. BMC Pregnancy Childbirth. (2019) 19(1):458. 10.1186/s12884-019-2617-8 31796036 PMC6889359

[7] Wu P Green M Myers JE. Hypertensive disorders of pregnancy. Br Med J. (2023) 381:e071653. 10.1136/bmj-2022-071653 37391211

[8] World Health Organization. Maternal Health. Geneva: WHO (2024). Available online at: http://www.who.int/topics/maternal_health/en/

[9] Wu P Haththotuwa R Kwok CS Babu A Kotronias RA Rushton C Preeclampsia and future cardiovascular health: a systematic review and meta analysis. Circ Cardiovasc Qual Outcomes. (2017) 10:e003497. 10.1161/CIRCOUTCOMES.116.003497 28228456

[10] Lawn JE Blencowe H Waiswa P Amouzou A Mathers C Hogan D Stillbirths: rates, risk factors and potential for progress towards 2030. Lancet. (2016) 387(10018):7. 10.1016/S0140-6736(15)00837-5 26794078

[11] Noubiap JJ Bigna JJ Nyaga UF Jingi AM Kaze AD Nansseu JR The burden of hypertensive disorders of pregnancy in Africa: a systematic review and meta-analysis. J Clin Hypertens. (2019) 21(4):479–88. 10.1111/jch.13514 PMC8030504 30848083

[12] Meazaw MW Chojenta C Muluneh MD Loxton D. Factors associated with hypertensive disorders of pregnancy in Sub-Saharan Africa: a systematic and meta-analysis. PLoS One. (2020) 15(8):e0237476. 10.1371/journal.pone.0237476 32813709 PMC7437911

[13] Firoz T Sanghvi H Merialdi M Von DP. Pre-eclampsia in low and middle income countries. Best Pr Res Clin Obs Gynaecol. (2011) 25:12. 10.1016/j.bpobgyn.2011.04.002 21592865

[14] Mahande JM. Risk factors for preterm birth among women who delivered preterm babies at Bugando Medical Centre, Tanzania. SOJ Gynecol Obstetr Womens Health. (2017) 3(2):7. 10.15226/2381-2915/3/2/00124

[15] Say L Chou D Gemmill A Tunçalp Ö Moller AB Daniels J Global causes of maternal death: a WHO systematic analysis. Lancet Glob Health. (2014) 2:e323–33. 10.1016/S2214-109X(14)70227-X25103301 25103301

[16] Ngwenya S. Severe preeclampsia and eclampsia: incidence, complications, and perinatal outcomes at a low-resource setting, Mpilo central hospital, Bulawayo, Zimbabwe. Int J Womens Health. (2017) 9:5. 10.2147/IJWH.S131934 PMC5439934 28553148

[17] Fekadu GA Kassa GM Berhe AK Muche AA Katiso NA. The effect of antenatal care on use of institutional delivery service and postnatal care in Ethiopia: a systematic review and meta-analysis. BMC Health Serv Res. (2018) 18(1):11. 10.1186/s12913-018-3370-9 30041655 PMC6056996

[18] Jaleta DD Gebremedhin T Jebena MG. Perinatal outcomes of women with hypertensive disorders of pregnancy in Jimma medical center, southwest Ethiopia: retrospective cohort study. PLoS One. (2021) 16(8):e0256520. 10.1371/journal.pone.0256520 34411170 PMC8375998

[19] FMOH. National Antenatal Care Guideline; Ensuring Positive Pregnancy Experience! Geneva: FMOH (2022).

[20] Syoum FH Abreha GF Teklemichael DM Chekole MK. Fetomaternal outcomes and associated factors among mothers with hypertensive disorders of pregnancy in Suhul hospital, northwest Tigray, Ethiopia. Hindawi J Pregnancy. (2022). 10.1155/2022/6917009 PMC9668464 36406161

[21] Berhe AK Ilesanmi AO Aimakhu CO Bezabih AM. Awareness of pregnancy induced hypertension among pregnant women in Tigray regional state, Ethiopia. Pan Afr Med J. (2020) 35(71). 10.11604/pamj.2020.35.71.19351 32537074 PMC7250223

[22] Berhe AK Kassa GM Fekadu GA Muche AA. Prevalence of hypertensive disorders of pregnancy in Ethiopia: a systemic review and meta-analysis. BMC Pregnancy Childbirth. (2018) 18(34). 10.1186/s12884-018-1667-7 PMC5774029 29347927

[23] Kassa BG Asnkew S Ayele AD Nigussie AA Demilew BC Mihirete GN. Preeclampsia and its determinants in Ethiopia: a systematic review and meta-analysis. PLoS One. (2023) 18(11):e0287038. 10.1371/journal.pone.0287038 37963147 PMC10645334

[24] Tesfa E Nibret E Gizaw ST Zenebe Y Mekonnen Z Assefa S Prevalence and determinants of hypertensive disorders of pregnancy in Ethiopia: a systematic review and meta-analysis. PLoS One. (2020) 15(9):e0239048. 10.1371/journal.pone.0239048 32936834 PMC7494091

[25] Getaneh T Negesse A Dessie G Desta M. The impact of pregnancy induced hypertension on low birth weight in Ethiopia: systematic review and meta-analysis. Ital J Pediatr. (2020) 46(174). 10.1186/s13052-020-00926-0 33243285 PMC7690116

[26] Page MJ McKenzie JE Bossuyt PM Boutron I Hoffmann TC Mulrow CD The PRISMA 2020 statement: an updated guideline for reporting systematic reviews. Br Med J. (2021) 372. 10.1186/s13643-021-01626-4 PMC8005924 33782057

[27] Van Zuuren E Fedorowicz Z. Moose on the Loose: Checklist for Meta-analyses of Observational Studies. Oxford, UK: Blackwell Publishing Ltd. (2016). p. 853–4. 10.1111/bjd.15038 27790686

[28] ACOG committee Opinion No 767. Emergent therapy for acute-onset, severe hypertension during pregnancy and the postpartum period. Obstet Gynecol. (2019) 133:409e12. 10.1097/AOG.0000000000003075 30575639

[29] Gaym A Bailey P Pearson L Admasu K Gebrehiwot Y. Disease burden due to pre-eclampsia/eclampsia and the Ethiopian health system’s response. Int J Gynecol Obstet. (2011) 115(1):112–6. 10.1016/j.ijgo.2011.07.012 21849170

[30] Davey D MacGillivray I. The classification and definition of the hypertensive disorders of pregnancy: proposals submitted to the international society for the study of hypertension in pregnancy. Clin Exp Hypertens B. (1986) 5(1):97–133. 10.3109/10641958609023478

[31] Magee LA Brown MA Hall DR Gupte S Hennessy A Karumanchi SA The 2021 international society for the study of hypertension in pregnancy classification, diagnosis & management recommendations for international practice. Pregnancy Hypertens 2022;27:148–69. 10.1016/j.preghy.2021.09.008 35066406

[32] Shea BJ Grimshaw JM Wells GA Boers M Andersson N Hamel C Development of AMSTAR: a measurement tool to assess the methodological quality of systematic reviews. BMC Med Rese Methodol. (2007) 7:7. 10.1186/1471-2288-7-10 PMC1810543 17302989

[33] Guyatt GH Oxman AD Vist GE Regina R Falck-Ytter Y Alonso-Coello P GRADE: an emerging consensus on rating quality of evidence and strength of recommendations. Br Med J. (2008) 336(7650):924–6. 10.1136/bmj.39489.470347.AD 18436948 PMC2335261

[34] Huedo-Medina TB Sánchez-Meca J Marín-Martínez F Botella J. Assessing heterogeneity in meta-analysis: q statistic or I2 index? Psychol Methods. (2006) 11(2):193. 10.1037/1082-989X.11.2.193 16784338

[35] Valentine JC Pigott TD Rothstein HR. How many studies do you need? A primer on statistical power for meta-analysis. J Educ Behav Stat. (2010) 35(2):215–47. 10.3102/1076998609346961

[36] Gudeta TA. Pregnancy induced hypertension and associated factors among pregnant women receiving antenatal care service at Jimma Town public health facilities, south west Ethiopia. J Gynecol Womens Health. (2018) 10(4):9. 10.4314/ejhs.v29i1.4

[37] Belay AS Wudad T. Prevalence and associated factors of pre-eclampsia among pregnant women attending anti-natal care at Mettu Karl referal hospital, Ethiopia: cross-sectional study. Clin Hypertens. (2019) 25(14). 10.1186/s40885-019-0120-1 31304042 PMC6600877

[38] Hinkosa L. Risk factors associated with hypertensive disorders in pregnancy in Nekemte referral hospital, from July 2015 to June 2017, Ethiopia: case control study. BMC Pregnancy Childbirth. (2017) 9:9. 10.1186/s12884-019-2693-9 PMC6945641 31906884

[39] Legesse AY Berhe Y Mohammednur SA Teka H. Prevalence and determinants of materna land perinatal outcome of preeclampsia at a tertiary hospital in abstract introduction. Ethiop J Reprod Health. (2019) 11(4):8. 10.69614/ejrh.v11i4.340

[40] Wodajo S Reddy PS. Hypertensive disorders of pregnancy and associated factors among admitted pregnant cases in Dessie town referral hospital, north east Ethiopia, 2015. Med Res. (2016) 39(1):77–90.

[41] Mekie M Mekonnen W Assegid M. Cohabitation duration, obstetric, behavioral and nutritional factors predict preeclampsia among nulliparous women in west Amhara Zones of Ethiopia: age matched case control study. PLoS One. (2020) 15(1):11. 10.1371/journal.pone.0228127 PMC6984729 31986179

[42] Hailu A Kebede D. Determinants of preeclampsia and gestational hypertension. Ethiop J Health Dev. (1991) 5(1):11. 10.2147/IJWH.S251342

[43] Aklilu A Abeje G Azage M Asmer S. Does Anemia Risk for Pre-Eclampsia?Amulticenter, Casecontrol Study in Amhararegion, Ethiopia. Preprint (2019).

[44] Tessema GA Tekeste A Ayele TA. Preeclampsia and associated factors among pregnant women attending antenatal care in Dessie referral hospital, northeast Ethiopia: a hospital-based study. BMC PregnancyChildbirth. (2015) 15(1):73. 10.1186/s12884-015-0502-7 PMC4392792 25880924

[45] Shegaze M Markos Y Estifaons W Taye L. Magnitude and associated factors of preeclampsia among pregnant women who attend antenatal care service in public health institutions in Arba Minch town, southern Ethiopia. Gynecol Obstetr. (2016) 6(12). 10.4172/2161-0932.1000419

[46] Vata PK Chauhan NM Nallathambi A Hussein F. Assessment of prevalence of preeclampsia from dilla region of Ethiopia. BMC Res Note. (2015) 8(1). 10.1186/s13104-015-1821-5 PMC4690301 26704295

[47] Seyom E Abera M Tesfaye M Fentahun N. Maternal and feta loutcome of pregnancy related hypertension in Mettu Karl referral hospital. Ethiopia J Ovarian Res. (2015) 8(1):10. 10.1186/s13048-015-0135-5 25824330 PMC4374296

[48] Obsa MS Wolka E. Maternal outcome of pregnant mothers with hypertensive disorder of pregnancy at hospitals in Wolaita zone, southern Ethiopia. J Preg Child Health. (2018) 5(375). 10.4172/2376-127X.1000375

[49] Wolde Z Segni H Woldie M. Hypertensive disorders of pregnancy in Jimma university specialized hospital. Ethiop J Health Sci. (2011) 21(3):147–54. 22434994 PMC3275872

[50] Terefe W Getachew Y Hiruye A Derbew M HaileMariam D Mammo D. Patterns of hypertensive disorders of pregnancy and associated factors in Debre Berhan referral hospital, north Shoa, Amhara region. Ethiop Med J. (2015):57–65. 26591284

[51] Selamawit D Sisay T. Maternal and perinatal outcomes of pregenanciescomplecated by preeclampsia/eclampsia at Zewditu memorial hospital. Medicine AAASoGSFo [Preprint]. Addis Ababa Aniversity (2015).

[52] Bilano VL Ota E Ganchimeg T Mori R Souza JP. Risk factors of pre-eclampsia/eclampsia and its adverse outcomes in low- and middle-income countries: a WHO secondary analysis. PLoS One. (2014) 9(3). 10.1371/journal.pone.0091198 24657964 PMC3962376

[53] Aromataris E Fernandez R Godfrey CM Holly C Khalil H Tungpunkom P. Summarizing systematic reviews: methodological development, conduct and reporting of an umbrella review approach. JBI Evid Implement. (2015) 13(3):132–40. 10.1097/XEB.0000000000000055 26360830

[54] Umesawa M Kobashi G. Epidemiology of hypertensive disorders in pregnancy: prevalence, risk factors, predictors and prognosis. Hypertens Res. (2017) 40(3):213–20. 10.1038/hr.2016.126 27682655

[55] Ye C Ruan Y Zou L Li G Li C Chen Y The 2011 survey on hypertensive disorders of pregnancy (HDP) in China: prevalence, risk factor s, complications, pregnancy and perinatal outcomes. PLoS One. (2014) 9(6). 10.1371/journal.pone.0100180 PMC4061123 24937406

[56] Naderi S Tsai SA Khandelwal A. Hypertensive disorders of pregnancy. Curr Atheroscler Rep. (2017) 19(3):15. 10.1007/s11883-017-0648-z 28229431

[57] Kuklina EV Ayala C Callaghan WM. Hypertensive disorders and severe obstetric morbidity in the United States. Obstet Gynecol. (2009) 113(6):1299–306. 10.1097/AOG.0b013e3181a45b25 19461426

[58] Algert CS Roberts CL Shand AW Morris JM Ford JB. Seasonal variation in pregnancy hypertension is correlated with sunlight intensity. Am J Obstet Gynecol. (2010) 203(3):215.e1–e5. 10.1016/j.ajog.2010.04.020 20537304

[59] Lazo-Vega L Toledo-Jaldin L Badner A Barriga-Vera JL Castro-Monrroy M Euser AG ACOG and local diagnostic criteria for hypertensive disorders of pregnancy (HDP) in La Paz-el Alto, Bolivia: a retrospective case-control study. Lancet Reg Health Am. (2020) 1(9). 10.1016/j.lana.2022.100194 PMC9205446 35719175

[60] Simkhada B Teijlingen ER Porter M Simkhada P. Factors affecting the utilization of antenatal care in developing countries: systematic review of the literature. J Adv Nurs. (2008) 61(3):244–60. 10.1111/j.1365-2648.2007.04532.x 18197860

[61] Yaya S Bishwajit G Ekholuenetale M Shah V Kadio B Udenigwe O. Timing and adequate attendance of antenatal care visits among women in Ethiopia. PLoS One. (2017) 12(9):e0184934. 10.1371/journal.pone.0184934 28922383 PMC5602662

[62] Manzi A Nyirazinyoye L Ntaganira J Magge H Bigirimana E Mukanzabikeshimana L Beyond coverage: improving the quality of antenatal care delivery through integrated mentorship and quality improvement at health centers in rural Rwanda. BMC Health Serv Res. (2018) 18(1):136. 10.1186/s12913-018-2939-7 29471830 PMC5824606

[63] Logan GG Njoroge PK Nyabola LO Mweu MM. Determinants of preeclampsia and eclampsia among women delivering in county hospitals in Nairobi, Kenya. F1000Res. (2020) 9:192. 10.12688/f1000research.21684.1

[64] Cunningham FG Leveno KJ Bloom SL Spong CY Dashe JS Hoffman BL Obstetrícia de Williams-25. Stamford, CT: Appleton & Lange Collection (2021). Available online at: https://www.ptonline.com/articles/how-to-get-better-mfi-results

[65] Jim B Karumanchi SA. Preeclampsia: pathogenesis, prevention, and long-term complications. Semin Nephrol (2017) 37(4):386–97. 10.1016/j.semnephrol.2017.05.011 28711078

[66] Kannel WB Vasan RS. Is age really a non-modifiable cardiovascular risk factor? Am J Cardiol. (2009) 104:1307–10. 10.1016/j.amjcard.2009.06.051 19840582 PMC3760670

[67] Meazaw MW Chojenta C Muluneh MD Loxton D. Systematic and meta-analysis of factors associated with preeclampsia and eclampsia in Sub-Saharan Africa. PLoS One. (2020) 15(8):1–23. 10.1371/journal.pone.0237600 PMC7437916 32813750

[68] Adu-Bonsaffoh K Ntumy MY Obed SA Seffah JD. Perinatal outcomes of hypertensive disorders in pregnancy at a tertiary hospital in Ghana. BMC Pregnancy Childbirth. (2017) 17(1):388. 10.1186/s12884-017-1575-2 29157196 PMC5696910

[69] Su Y-Y Zhang J-Z Wang F. Risk factors and adverse outcomes of preeclampsia: a tertiary care centrebased study in China. Biomed Res. (2017) 28(3):1262–5. 10.21203/rs.3.rs-3829930

[70] Amaral LM Wallace K Owens M LaMarca B. Pathophysiology and current clinical management of preeclampsia. Curr Hypertens Rep. (2017) 19(8):61. 10.1007/s11906-017-0757-7 28689331 PMC5916784

[71] Tai M Piskorski A Kao JCW Hess LA de la Monte SM Gündoğan F. Placental morphology in fetal alcohol spectrum disorders. Alcohol Alcohol (2017) 52(2):138–44. 10.1093/alcalc/agw088 28182213 PMC6248725

[72] Iwama N Metoki H Nishigori H Mizuno S Takahashi F Tanaka K Association between alcohol consumption during pregnancy and hypertensive disorders of pregnancy in Japan: the Japan environment and children’s study. Hypertens Res. (2019) 42(1):85–94. 10.1038/s41440-018-0124-3 30401907

[73] Al-Motarreb A Al-Habori M Broadley KJ. Khat chewing, cardiovascular diseases and other internal medical problems: the current situation and directions for future research. J Ethnopharmacol. (2010) 132:540–8. 10.1016/j.jep.2010.07.001 20621179

[74] Mekonen L Shiferaw Z Wubshet E Haile S. Pregnancy induced hypertension and associated factors among pregnant women in Karamara hospital, Jijiga, eastern Ethiopia. J Pregnancy Child Health. (2018) 4(2):831–40. 10.4172/2376-127X.1000379

