# Supplementary material for: The impact of hypertensive disorders of pregnancy on maternal and perinatal outcomes in Ethiopia: an umbrella review of systematic reviews
Source: Front Glob Womens Health. 2025 Jul 21;6:1571052. doi: 10.3389/fgwh.2025.1571052 (PMC12319007; doi:10.3389/fgwh.2025.1571052)
Supplement: Supplementary file 1 [file Table1.docx]

Supplementary table1; MOOSE checklist on the impacts of hypertensive disorders of pregnancy on maternal and perinatal outcomes in Ethiopia

| Criteria | | Brief description of how the criteria were handled in  the meta-analysis |
| --- | --- | --- |
| **Reporting of background should**  **include** | |  |
| 1 | Problem definition | Page 3: Although previous meta-analyses and systematic reviews (SRMAs) have examined this issue, the variability in the findings poses challenges for healthcare initiatives and medical management.  Furthermore, the evidence has not been synthesized in a manner that makes it accessible for addressing the factors related to HDP. |
| 2 | Hypothesis statement | Page:3 In Ethiopia, HDP is the second leading cause of maternal fatalities, premature births, and perinatal mortality as indicate by the 2020 report on maternal perinatal death surveillance and response identified  The occurrence of HDP in Ethiopia varies significantly, with reported rates between 2.3% and 64.1% as indicated by several studies. Additionally, the prevalence of pre-eclampsia is reported to be between 1.2% and 19.1%. |
| 3 | Description of study outcomes | Page 4: magnitude, prevalence, determinants, predictors, associated factors, correlates, risk factors, adverse effects of hypertensive disorders of pregnancy, complications of hypertensive disorders of pregnancy, and consequences of hypertensive disorders of pregnancy on maternal and perinatal outcomes in Ethiopia |
| 4 | Type of exposure or intervention used | Page 4: Normotensive pregnant women |
| 5 | Type of study designs used | Page 4: SRMAs. |
| 6 | Study population | Page 5: pregnant women having HDP |
| **Reporting of search strategy should include** | |  |
| 7 | Qualifications of searchers | Page 4: The credentials of the two investigators  TEG and SA are indicated in the author list. |
| 8 | Search strategy, including time period included in the synthesis and keywords | Page 4: The search was conducted from July 15 to 25, 2024. Key words include; Hypertensive disorders of pregnancy, Preeclampsia, Eclampsia, Outcomes, Pregnant women, Systemic review, Meta -analysis |
| 9 | Databases and registries searched | Page 4: MEDLINE/PubMed, Cochrane, Web of Science and Science Direct |
| 10 | Search software used, name and version, including special features | Page 5: We have not used search software. But, the Endnote citation manager (version X8, for Windows; Thomson Reuters, Philadelphia, PA, USA) was applied to import the retrieved studies. |
| 11 | Use of hand searching | Pages 5: We hand-searched bibliographies of retrieved papers for additional references |
| 12 | List of citations located and those excluded, including justifications | Pages 4-5: Details of the literature search process are outlined in supplementary file. |
| 13 | Method of addressing articles published in languages other than English | Page 5: We limited to the studies published in English |
| 14 | Method of handling abstracts and unpublished studies | Page 5: We have not included unpublished studies on our umbrella review |
| 15 | Description of any contact with authors | Not applicable |
| **Reporting of methods should include** | |  |
| 16 | Description of relevance or appropriateness of studies assembled for assessing the hypothesis to be tested | Pages 5: Detailed inclusion and exclusion criteria were described in the study selection section. |
| 17 | Rationale for the selection and coding of data | Page 5: Data extracted from each of the studies were relevant to the first author, study characteristics, characteristics of participants, outcome characteristics. |
| 18 | Assessment of confounding | Not applicable |
| 19 | Assessment of study quality, including blinding of quality assessors; stratification or regression on possible predictors of study results | Page 6: Each study in the analysis underwent a thorough evaluation using the Assessment of Multiple Systematic Reviews (AMSTAR) tool |
| 20 | Assessment of heterogeneity | Page 6: Higgins' I2 statistics were used to determine the degree of heterogeneity between studies, which guided the choice of meta-analysis methodology. According to Higgins et al, I2 < 49%, 50–75, and > 75%, respectively, indicate low, moderate, and high degrees of heterogeneity. Because of the substantial variations within and between studies, the random-effects model was used to produce the pooled prevalence estimates |
| 21 | Description of statistical methods in sufficient detail to be replicated | Pages 6: Description of methods of meta-analyses was detailed in the data synthesis and analysis section. |
| 22 | Provision of appropriate tables and graphics | We included 1 flow chart, 3 summary tables, 6  Figures and 2 supplementary files |
| **Reporting of results should include** | |  |
| 23 | Graph summarizing individual study estimates and overall estimate | Figure 2 |
| 24 | Table giving descriptive information for each study included | Table 1 |
| 25 | Results of sensitivity testing | Not applicable |
| 26 | Indication of statistical uncertainty of finding | Pages 10 95% confidence intervals were presented with all summary estimates. |
| **Reporting of discussion should include** | | |
| 27 | Quantitative assessment of bias | Not applicable since studies were less than 10. |
| 28 | Justification for exclusion | Pages 5: Studies were excluded if they met any of the following criteria:  No report on the prevalence or determinants of HDP or complications of, narrative reviews editorials, correspondence, abstracts, and methodological studies. literature reviews that lacked a defined research question, search strategy, or article selec­tion process were excluded |
| 29 | Assessment of quality of included studies | Using the AMSTAR tool the methodological quality of included SRM studies evaluated. The quality of scoring was done out of 11 points and ranged from 8 to 10 (**Table 3**). |
| **Reporting of conclusions should include** | |  |
| 30 | Consideration of alternative explanations for observed results | Page 11: We discussed the limitations of this study. |
| 31 | Generalization of the conclusions | Page 11: The higher rate of severe forms of HDP that are associated with significant maternal and perinatal complications is a major concern in the Ethiopia. The risk of developing HDP is worse among women, who have a history of preeclampsia, maternal age >35 years, alcohol consumption and its complications such as low birth weight. |
| 32 | Guidelines for future research | Promotion of early antenatal care (ANC) attendance through existing platforms, raising awareness about critical warning signs during pregnancy, avoidance of alcohol consumption, ensuring timely referrals to nearby health facilities, and strengthen maternity waiting homes should also be organized and supported.. |
| 33 | Disclosure of funding source | No Applicable |
