# Supplementary material for: The impact of hypertensive disorders of pregnancy on maternal and perinatal outcomes in Ethiopia: an umbrella review of systematic reviews
Source: Front Glob Womens Health. 2025 Jul 21;6:1571052. doi: 10.3389/fgwh.2025.1571052 (PMC12319007; doi:10.3389/fgwh.2025.1571052)
Supplement: Supplementary file 2 [file Table2.docx]

Supplementary Table 2: Search Strategy in different databases on the impacts of hypertensive disorders of pregnancy on maternal and perinatal outcomes in Ethiopia

| Data base | Search | Query | Search results* |
| --- | --- | --- | --- |
| PubMed | #1 | Search: ((Hypertensive Disorders of pregnancy and associated factors in Ethiopia) OR (Maternal and perinatal outcomes of hypertensive disorders of pregnancy and associated factors in Ethiopia)) OR (the Impacts of Hypertensive Disorders of Pregnancy on Maternal and Perinatal Birth Outcomes in Ethiopia systematic review and meta-analysis) | 72 |
|  | #2 | Search:  ((((((((((((((((((((((prevalence) OR (magnitude)) OR (determinants)) OR (impacts)) OR (predictors)) AND (factors)) OR (risk factors)) OR (associated factors))) AND (fetal outcomes)) OR (maternal outcome)) OR (infant)) OR (neonatal)) OR (LBW)) OR (very low birth weight)) OR (adverse outcomes)) OR (underweight)) OR (birth outcome)) OR (mother)) AND ((Hypertension))) OR (Pregnancy)) OR (Pregnancy induced hypertension)) OR (gestational hypertension)) OR (Pre- Eclampsia)) OR (hypertensive disorder of pregnancy)) AND (Ethiopia) Filters: Meta-Analysis, Systematic Review | 19 |
|  | #3 | Search: ((Hypertensive Disorders of pregnancy and associated factors in Ethiopia) OR (Maternal and perinatal outcomes of hypertensive disorders of pregnancy and associated factors in Ethiopia)) OR (the impacts of hypertensive disorders of pregnancy on maternal and perinatal birth outcomes in Ethiopia systematic review and meta-analysis) AND (systematic review)) OR (meta-analysis)) AND (Ethiopia) Filters: Meta-Analysis, Systematic Review | 11 |
|  | #4 | Search:  ((((((((((((((((((((((prevalence) OR (magnitude)) OR (determinants)) OR (impacts)) OR (predictors)) AND (factors)) OR (risk factors)) OR (associated factors))) AND (fetal outcomes)) OR (maternal outcome)) OR (infant)) OR (neonatal)) OR (LBW)) OR (very low birth weight)) OR (adverse outcomes)) OR (underweight)) OR (birth outcome)) OR (mother)) AND ((Hypertension))) OR (Pregnancy)) OR (Pregnancy induced hypertension)) OR (gestational hypertension)) OR (Pre- Eclampsia)) OR (hypertensive disorder of pregnancy)) AND (Ethiopia) AND (meta-analysis[Filter] OR review[Filter] OR systematic review [Filter])Filters: Meta-Analysis, Systematic Review | 8 |
|  | #5 | #1 AND #2 AND #3AND#4 | 2 |
| Research 4 life | #1 | The Impacts of Hypertensive Disorders of Pregnancy on Maternal and Perinatal Birth Outcomes in Ethiopia: An Umbrella Review of Systematic Reviews | 4 |
| PsycINFO | #1 | Hypertensive Disorders of pregnancy and associated factors, maternal and perinatal outcomes of hypertensive disorders of pregnancy and associated factors, the impacts of hypertensive disorders of pregnancy on maternal and perinatal birth outcomes in Ethiopia: (review article) | 6 |
| Cochrane library | #1 | Cochrane Reviews matching prevalence in Title Abstract Keyword OR magnitude in Title Abstract Keyword AND determinants in Title Abstract Keyword OR risk factors in Title Abstract Keyword AND The Impacts of Hypertensive Disorders of Pregnancy on Maternal and Perinatal Birth Outcomes in Ethiopia in Title Abstract Keyword - (Word variations have been searched) | 7 |
| CINAHL | #1 | the impacts of hypertensive disorders of pregnancy on maternal and perinatal birth outcomes in Ethiopia: review article | 5 |
| Science Direct | #1 | Prevalence and determinants of hypertensive disorders of pregnancy on maternal and perinatal birth outcomes in Ethiopia: (review article) | 8 |
| Other data base (goggle scholar and Africa journal online | #1 | Prevalence and determinants of hypertensive disorders of pregnancy on maternal and perinatal birth outcomes in Ethiopia: systematic review and meta-analysis | 14 |

**= Date of search: from September*  15 to 25, 2024
