# Supplementary material for: The impact of hypertensive disorders of pregnancy on maternal and perinatal outcomes in Ethiopia: an umbrella review of systematic reviews
Source: Front Glob Womens Health. 2025 Jul 21;6:1571052. doi: 10.3389/fgwh.2025.1571052 (PMC12319007; doi:10.3389/fgwh.2025.1571052)
Supplement: Supplementary file 3 [file Table3.docx]

| **Number of Studies** | **Sample size** | **Prevalence with 95% CI** | **Pooled estimate** | **P value** | **Heterogenety** | **Weight** |
| --- | --- | --- | --- | --- | --- | --- |
| 2 | <10,000 | 19.7 (4.25, 25.5) | 27.7 (18.81, 30.49) | 0.000 | 90.05 | 19.16 |
|  |  | 39.7 (33.3, 46.2) |  | 0.000 | 89.4 | 20.17 |
| 3 | >10,000 | 5.78 (4.95, 6.62) | 5.43 (7.50, 9.62) | 0.000 | 92.5 | 20.00 |
|  |  | 11.51(8.41, 14.61) |  | 0.000 | 99.5 | 19.53 |
|  |  | 6.07 (4.83, 7.31) |  | 0.000 | 99.4 | 21.14 |
|  |  |  | 16.56 (13.15, 20.0) |  | 94.17 | 100 |

Subgroup analyses for the prevalence of impacts of HDP among Ethiopian pregnant woman, 2024

Obtained from the Random-effects model
