# Supplementary material for: The impact of hypertensive disorders of pregnancy on maternal and perinatal outcomes in Ethiopia: an umbrella review of systematic reviews
Source: Front Glob Womens Health. 2025 Jul 21;6:1571052. doi: 10.3389/fgwh.2025.1571052 (PMC12319007; doi:10.3389/fgwh.2025.1571052)
Supplement: Supplementary file 4 [file Table4.docx]

| **Study omitted** | **Pooled estimate** | **95%CI** |
| --- | --- | --- |
| Mersha et al. | 15.84 | (13.19-19.48) |
| Berhe et al. | 17.87 | (14.08-19.76) |
| Getaneh et al. | 16.56 | (13.11-20.02) |
| Tesfa et al. | 16.89 | (12.23-20.64) |
| Kassa et al. | 15.66 | (13.18- 20.13) |
| Total | 16.56 | (13.15, 20.02) |

**Table 4** Sensitivity analyzes for the pooled prevalence of impacts of hypertensive disorders of pregnancy among pregnant women in Ethiopia, 2024
